# Oral Care of Post-Stroke Dysphagia Patients: A Cross-Sectional Survey of Neurological Nurses’ Knowledge, Attitudes, and Practices

**DOI:** 10.3290/j.ohpd.c_2862

**Published:** 2026-11-11

**Authors:** Xiaoling Hu, Shijun Li, Juan Chen, Chunlian Liao, Menglu Zhong

**Affiliations:** a Xiaoling Hu Associate Chief Nurse, Department of Neurology, Wuxi Country People’s Hospital of Chongqing, Chongqing, 405800, China; responsible for the research design and writing of the first draft; b Shijun Li Chief Nurse, Department of Neurology, Wuxi Country People’s Hospital of Chongqing, Chongqing, 405800, China; responsible for reviewing and editing the manuscript; c Juan Chen Chief Nurse, Department of Neurology, Wuxi Country People’s Hospital of Chongqing, Chongqing, 405800, China; responsible for the data statistics and analysis; d Chunlian Liao Chief Nurse, Department of Neurology, The Second Affiliated Hospital of Chongqing Medical University, Chongqing, 405800, China; responsible for reviewing and editing the manuscript; e Menglu Zhong Supervisor Nurse, Department of Neurology, Wuxi Country People’s Hospital of Chongqing, Chongqing, 405800, China; responsible for the investigation

**Keywords:** knowledge-attitude-practice, nurses, oral care, post- stroke dysphagia

## Abstract

**Objective:**

Most post-stroke dysphagia (PSD) patients suffer from compromised oral health, which necessitates standardised oral care delivered by nurses. This study aimed to evaluate neurological nurses’ knowledge, attitudes, and practices (KAP) related to oral care for PSD patients and explore associated influencing factors.

**Materials and methods:**

A cross-sectional survey was performed from September to November 2024 with convenience sampling across Southwest China. Data were gathered via an online questionnaire consisting of two parts: demographic characteristics and a self-developed KAP scale tailored for neurological nurses responsible for oral care of PSD patients. Univariate correlation analysis and multiple linear regression analysis were applied to identify predictors of KAP scores.

**Results:**

In total, 337 valid questionnaires were retrieved and analysed, corresponding to an effective response rate of 92%. Around 70.92% (n = 239) of participating nurses had never received targeted training on oral care for PSD patients. The mean total KAP score for neurological nurses regarding oral care for PSD patients was 121.45 ± 18.15, with scores for knowledge, attitudes, and practices being 53.98 ± 10.96, 43.75 ± 7.14, and 23.82 ± 6.50, respectively. Multiple linear regression demonstrated that hospital level, educational attainment, years of neurological nursing experience, and prior specialised training were significant predictors of KAP total scores (*P* < 0.05).

**Conclusion:**

Neurological nurses had deficiencies in all three KAP dimensions for PSD oral care. Standardised targeted training programmes should be developed to improve nurses’ professional oral care competence.

Post-stroke dysphagia (PSD) commonly impairs oral functions including mastication and ingestion, which negatively affects the oral health of nearly 80% of stroke survivors.^15^ Most stroke survivors are older adults, and oral frailty manifestations such as periodontitis, dental caries, sialorrhoea, and xerostomia are highly prevalent among those living with dysphagia.^12^ In a previous investigation, Wu et al^27^ identified dental caries (91.7%, 278/303) and poor oral hygiene (92.2%, 319/346) as the predominant oral problems affecting PSD patients, both of which greatly compromised their oral health–related quality of life (OHRQoL). Likewise, Tian et al^24^ verified that PSD patients had worse oral health status than non-dysphagic stroke survivors, evidenced by lower scores on the Oral Health Assessment Tool (OHAT). Failure to implement standardised oral care procedures among PSD patients not only impairs patients’ comfort levels but also raises the risk of pathogenic oral microbial colonisation,^3^ which is strongly associated with stroke-associated pneumonia (SAP).^26^ Notably, elderly PSD patients with poor oral hygiene face substantially elevated risks of SAP and malnutrition. Furthermore, poor oral health caused by insufficient oral care delivery will aggravate the severity of various systemic disorders associated with stroke, such as myocardial infarction, recurrent stroke, diabetes mellitus, and Alzheimer’s disease.^11^


High-standard oral care is therefore critical to preserving oral health, lowering SAP incidence and facilitating the rehabilitation of swallowing function.^14^ Rehabilitation guidelines issued by China, South Korea, and the United Kingdom all underscore the role of standardised oral care in reducing SAP risk for PSD patients.^9,13,30
^ Nevertheless, oral care is frequently deprioritised by neurological nurses as it is not regarded as an urgent life-saving intervention. Multiple barriers limit the delivery of adequate oral care to this patient group. First, existing survey data indicate that 86.7% of stroke survivors with concurrent dysphagia and upper-extremity dysfunction cannot complete independent oral self-care due to physical limitations.^17^ Second, post-stroke cognitive dysfunction, depressive symptoms, and communication barriers complicate nurse-led oral care operations.^10^ Third, the absence of systematic clinical guidelines further aggravates this issue. Bangee et al^4^ pointed out that post-stroke oral care is plagued by missing unified protocols, non-standardised assessment tools, and inadequate staff training, resulting in inconsistent clinical practices across different hospitals. As a result, neurological nurses lack sufficient oral care knowledge, awareness, and practical skills, and this competency gap is especially prominent in underdeveloped regions. Southwest China is a region with relatively limited medical resources, where standardised oral care procedures for PSD patients may be insufficient. Against this background, the present study investigated the current oral care situation and analysed predictors of knowledge, attitudes, and practices (KAP) among neurological nurses managing PSD patients in Southwest China.

## Materials and methods

### Study Design

This cross-sectional survey was carried out between September and November 2024. Each questionnaire took participants 6 to 15 minutes to complete.

#### Settings and participants 

Convenience sampling was adopted to recruit nurses from 10 public hospitals in primary, secondary, and tertiary tiers across Southwest China, covering Chongqing, Sichuan, Yunnan, and Guizhou Provinces. Primary hospitals are community or township-level facilities providing basic preventive and routine medical services for local residents; secondary hospitals are county-level hospitals delivering comprehensive inpatient and outpatient care and undertaking limited teaching and research tasks; and tertiary hospitals are high-level referral centres (including prefecture-level, provincial, and university-affiliated hospitals) responsible for the treatment of complex and severe diseases, advanced medical education and scientific research. The inclusion criteria were nurses with a valid nursing qualification and those working in neurology departments and managing stroke patients with dysphagia, whereas the exclusion criteria were nurses on vacation or rotation and nursing students or those temporarily assigned for training from other institutions.

#### Sample size calculation

Based on the Kendall sample size rule (the sample size should reach 5 to 10 times the number of questionnaire items), the minimum sample size for this 36-item questionnaire was 160 to 320 participants. After accounting for a 10% expected non-response rate, the adjusted target sample size ranged from 176 to 352. A total of 366 nurses participated in the survey, which exceeded the calculated minimum sample size.

#### Questionnaire design

The questionnaire was specifically designed to assess the KAP status of neurological nurses regarding oral care for PSD patients. First, a chief nurse with over 15 years of stroke care experience and a nurse with a master’s degree conducted a comprehensive literature review and developed an initial 45-item KAP questionnaire.The questionnaire drew on the World Health Organization’s Global Strategy and Action Plan for Oral Health (2023–2030), Oral Health: Supporting Adults Who Require Assistance (2nd edition),^22^ and Chinese Dysphagia Rehabilitation Management Guidelines (2023 Edition).^9^ The questionnaire also referenced validated tools such as the 21-item questionnaire by Binkley et al,^5^ oral cleaning solutions and tools identified by Lin et al,^19^ and practices related to oral care in qualified nursing staff in medical wards, as studied by Adams.^1^ Collectively, these materials covered four core thematic modules: the causes and clinical manifestations of oral issues in PSD patients, oral assessments, oral cleaning solutions, and oral cleaning tools. Secondly, sixteen experts were invited for two rounds of Delphi expert consultations to validate and revise the questionnaire. The expert panel comprised two directors from neurological departments, two therapists from rehabilitation departments, one director from a dental department, and eleven head nurses from neurological departments. The authority coefficient (Cr) of Delphi expert consultation was 0.84; the mean item importance scores were 3.5 to 5.0 and 3.75 to 5.0 for the two rounds respectively. The Kendall W coefficients for the two rounds were 0.220 and 0.246, respectively, with *P* < 0.001. The coefficient of variation (CV) for the two rounds ranged from 0.10 to 0.32, and 0.07 to 0.21, respectively. During the revision process, eight items with a mean importance score < 4 and CV > 0.25 were deleted. Based on expert recommendations, several items were adjusted, split, or merged. Ultimately, the questionnaire consisted of 32 Likert-scale items and five multiple-choice questions, demographic information, and a knowledge dimension (14 items), attitude dimension (11 items), and practice dimension (7 items).

Before the formal survey, a pilot survey was conducted involving 40 nurses from the neurological departments of one secondary hospital and one tertiary hospital in Chongqing. This survey aimed to examine the reliability of the questionnaire, the accuracy of its language expression, and the time required for completion. Nurses who participated provided feedback on the logical structure and linguistic accuracy of the items. The item-level content validity index (I-CVI) for all items ranged from 0.88 to 1.00, and the scale-level content validity index (S-CVI) of the whole questionnaire was 0.98. The overall Cronbach α coefficient was 0.915. The Cronbach α values of the knowledge, attitude, and practice dimensions were 0.949, 0.915, and 0.881 respectively, demonstrating excellent internal consistency. The split-half reliability coefficient was 0.858, and the 2-week test-retest reliability coefficient was 0.853 (*P* < 0.05).

#### Sociodemographic and clinical data

The general information section covered participants’ sex, age, length of nursing service, hospital tier, department, professional title, monthly income, and prior training related to oral care for PSD patients. There were three sections: the knowledge dimension, attitudes dimension, and practices dimension section. The knowledge dimension section contained 14 items scored on a 5-point Likert scale (1, completely unfamiliar; 2, somewhat unfamiliar; 3, moderately familiar; 4, relatively familiar; 5, very familiar) and the total possible score ranged from 14 to 70. The attitudes dimension section included 11 items scored on a 5-point Likert scale (1, strongly disagree; 2, disagree; 3, neutral; 4, agree; 5, strongly agree) and the total possible score ranged from 11 to 55. The practices dimension section consisted of 12 items, 7 of which were rated on a 5-point Likert scale (1, never; 2, occasionally; 3, sometimes; 4, often; 5, always) and the total possible score ranged from 7 to 35. The other five unscored multiple-choice items investigated barriers to oral care delivery, routine oral care frequency, priority oral assessment indicators, frequently adopted oral cleaning solutions, and oral cleaning tools. These multiple-choice items carried no scoring weight and were only used to descriptively analyse hospital-level oral care resources and clinical practices.

#### Data collection

The survey was conducted online using the Questionnaire Star platform, accessible via WeChat. A link to the questionnaire, containing details on the study’s purpose, significance, and data privacy assurances, was distributed. Before filling out the questionnaire, all participants were presented with a detailed informed consent statement on the webpage, including the research purpose, data usage, privacy protection, and voluntary participation principles. Participants could only proceed to answer questions after actively clicking to agree to the electronic informed consent. No paper-based signed informed consent forms were collected in this survey. The questionnaire system automatically checked form completeness to guarantee data quality. Responses that failed the validity check were eliminated per preset exclusion standards. Double data entry was applied to ensure data accuracy, and each IP address was limited to one single submission to avoid duplicate responses.

#### Data analysis

All statistical analyses of survey data were performed with SPSS 27.0 (IBM, Armonk, NY, USA). Descriptive statistics (means, standard deviations, and constituent ratios) were calculated to summarise the KAP scores. The KAP dimension scores were set as dependent variables, whereas statistically significant sociodemographic variables were entered as independent variables. Stepwise multiple linear regression was used to identify predictors of total KAP scores. Pearson correlation analysis was also conducted to examine inter-variable associations and further interpret factors associated with KAP performance.

#### Ethical considerations

This study obtained ethical approval from the Ethics Committee of Wuxi County People’s Hospital, Chongqing (Approval No. 2024-93). All participants provided anonymous electronic informed consent in accordance with the Declaration of Helsinki.

## Results

### Participant Characteristics 

A total of 366 questionnaires were collected. Two exclusion criteria were applied to screen invalid responses: abnormal completion time (questionnaires finished in less than 150 seconds or more than 1500 seconds were eliminated, with the time threshold determined based on the completion duration recorded in the pilot survey) and duplicate submissions from the same IP address. After screening, 29 invalid questionnaires were removed, leaving 337 valid responses with an effective response rate of 92%.

The participants had a mean age of 32.5 ± 7.5 years, and 326 (96.74%) were female. In terms of hospital level, 243 (72.11%) worked in tertiary hospitals, 86 (25.52%) in secondary hospitals, and 8 (2.37%) in primary hospitals. A total of 239 (70.92%) nurses reported no previous specialised training on oral care for patients with PSD, while 168 (49.85%) had accumulated ≥ 10 years of neurological nursing experience. Additional demographic information is presented in Table 1.

**Table 1 Table1:** Demographic characteristics and overall KAP scores of participants (N = 337)

Item	Variable	n (%)
Hospital level	Primary	8 (2.37)
	Secondary	86 (25.52)
	Tertiary	243 (72.11)
Department	Neurological	153 (45.40)
	Neurosurgery	153 (45.40)
	Comprehensive	31 (9.20)
Sex	Male	11 (3.26)
	Female	326 (96.74)
Age (y)	< 30	39 (11.57)
	30~40	260 (77.15)
	> 40	38 (11.28)
Years of clinical practice in neurology	≤ 3	47 (13.95)
	4~6	61 (18.10)
	7~9	61 (18.10)
	≥ 10	168 (49.85)
Educational level	Junior college	51 (15.13)
	Bachelor’s degree and above	286 (84.87)
Position	Nurse	301 (89.32)
	Head nurse	36 (10.68)
Title	Nurse	54 (16.02)
	Nurse practitioner	122 (36.20)
	Head nurse and above	161 (47.77)
Monthly income (yuan)	< 6,000	181 (53.71)
	6,000~10,000	114 (33.83)
	> 10,000	42 (12.46)
Training	Yes	98 (29.08)
	No	239 (70.92)


#### Overall status of KAP of neurological nurses regarding oral care for PSD patients

The mean total KAP score for neurological nurses regarding oral care for PSD patients was 121.45 ± 18.15, with scores for knowledge, attitudes, and practices being 53.98 ± 10.96, 43.75 ± 7.14, and 23.82 ± 6.50, respectively, as shown in Table 2. In response to multiple-choice questions, 61.8% of nurses reported performing oral care for patients twice per day. Regarding oral assessments, 70% and 69% of nurses prioritised ‘oral odour’ and ‘oral mucosa’, respectively. Barriers to implementing oral care were attributed to ‘patient non-cooperation’ and ‘difficulty of oral care procedures’ by 67% and 54% of respondents, respectively. Moreover, 76.5% of nurses favoured normal saline as their primary oral cleaning solution, whereas 50.21% selected suction toothbrush as their main oral cleaning tool. For further details, see Fig 1.

**Table 2 Table2:** Mean KAP scores

Variable		Rank	Average score, mean ± SD
Knowledge	Total knowledge score	NA	53.98 ± 10.96
	K8. Do you know that the main purpose of sodium chloride mouth cleaning fluid, commonly used for PSD patients, is to remove oral debris and plaque?	1	4.10 ± 0.98
	K2. You know that the clinical manifestations of oral problems in PSD patients include salivation, dry mouth, tongue muscle weakness, food retention in the mouth, and oral infection.	2	4.07 ± 0.93
	K1. Do you know that good oral hygiene can prevent SAP in PSD patients?	3	3.99 ± 0.96
	K4. You know that PSD patients with a high-sugar diet and diabetes mellitus are more likely to have oral problems.	4	3.97 ± 0.96
	K12. You know that the oral assessment of PSD patients includes teeth/dentures, gingiva, saliva, tongue, mucous membranes, damage, odour, lips, soft palate, self-care ability, and health knowledge.	5	3.93 ± 0.96
	K3. You know that bacterial and fungal respiratory infections are easy to detect in the oral saliva samples of PSD patients.	6	3.91 ± 0.94
	K14. You know that oral care combined with oral sensory stimulation training can help PSD patients recover swallowing function.	7	3.90 ± 0.97
	K6. You know that the sodium bicarbonate oral cleaning solutions commonly used by PSD patients mainly play an antibacterial role, and the common concentration is 1%~4%.	8	3.86 ± 1.09
	K7. You know that the hydrogen peroxide oral cleaning solutions commonly used by PSD patients mainly play an antiseptic and deodorant role, and the common concentration is 1%~3%.	9	3.83 ± 1.08
	K9. You know that PSD patients should use fluoride toothpaste, toothbrush, dental floss, a negative pressure toothbrush, and other appliances as appropriate to clean their mouth after meals.	10	3.76 ± 1.12
	K11. You know that if a PSD patient has red and swollen gums, shrinking, bleeding, bad breath, long teeth, or even loose teeth, it may be a symptom of periodontal disease.	11	3.73 ± 1.09
	K13. You are aware that some medications used by PSD patients can cause dry mouth, such as antiepileptic drugs, antidepressants, and antihypertensive drugs.	12	3.70 ± 1.04
	K5. You know that the concentration of chlorhexidine oral cleaning solutions commonly used by PSD patients is 0.05~0.12%.	13	3.62 ± 1.11
	K10. You know that PSD patients should brush their teeth using the Bass brushing method: that is, the toothbrush is at a 45-degree angle to the teeth, the brim is aimed at the intersection of the gum and the teeth, and the teeth are brushed with a gentle, circular, up-and–down massage movement.	14	3.61 ± 1.20
Attitude	Total attitude score	NA	43.65 ± 7.14
	A9. Would you like to participate in training covering oral care knowledge and skills for PSD patients?	1	4.13 ± 0.78
	A10. Do you think more oral care training would enhance your competency in managing oral care for PSD patients?	2	4.12 ± 0.88
	A2. In your opinion, is oral care more easily neglected than disease assessment and rehabilitation procedures?	3	4.08 ± 0.81
	A3. In your opinion, should nurses be competent to clear dental plaque for PSD patients to avoid caries and periodontal disorders?	4	4.01 ± 0.81
	A8. Are you willing to proactively learn relevant knowledge about oral care for PSD patients?	5	4.01 ± 0.90
	A5. In your opinion, should dental practitioners join multidisciplinary teams for PSD management?	6	4.00 ± 0.89
	A7. In your opinion, is it vital to guide PSD patients and their families to perform oral motor and sensory training?	7	4.00 ± 0.92
	A11. In your opinion, are you competent to provide standard guidance on oral functional training for PSD patients?	8	3.99 ± 0.82
	A6. Do you think the department should formulate and update oral care plans?	9	3.93 ± 0.92
	A4. Do you think you know how to identify and prevent oral infections?	10	3.74 ± 0.93
	A1. Do you think oral care is very important for the overall rehabilitation of PSD patients?	11	3.64 ± 0.99
Practice	Total practice score	NA	23.82 ± 6.50
	P6. You will tailor your oral cleaning methods to the specific situation of the PSD patient.	1	3.69 ± 1.30
	P4. If you identify potential oral infection in a PSD patient during assessment, you will recommend oral secretion culture to the attending physician.	2	3.60 ± 1.29
	P2. You will use an oral assessment tool to assess the oral condition of the PSD patient.	3	3.50 ± 1.25
	P5. You will assess the risk of fluid aspiration before delivering oral care to PSD patients.	4	3.48 ± 1.32
	P7. You will instruct oral exercises (oral motor and sensory training) while performing oral care for PSD patients.	5	3.42 ± 1.40
	P3. If you assess the oral problems of PSD patients, you will report them to the doctor in charge and the dental practitioner in time.	6	3.40 ± 1.42
	P1. You will assess the oral condition of PSD patients on a daily basis.	7	2.72 ± 1.13
KAP			121.45 ± 18.15
NA, not applicable.			

**Fig 1a to e Fig1atoe:**
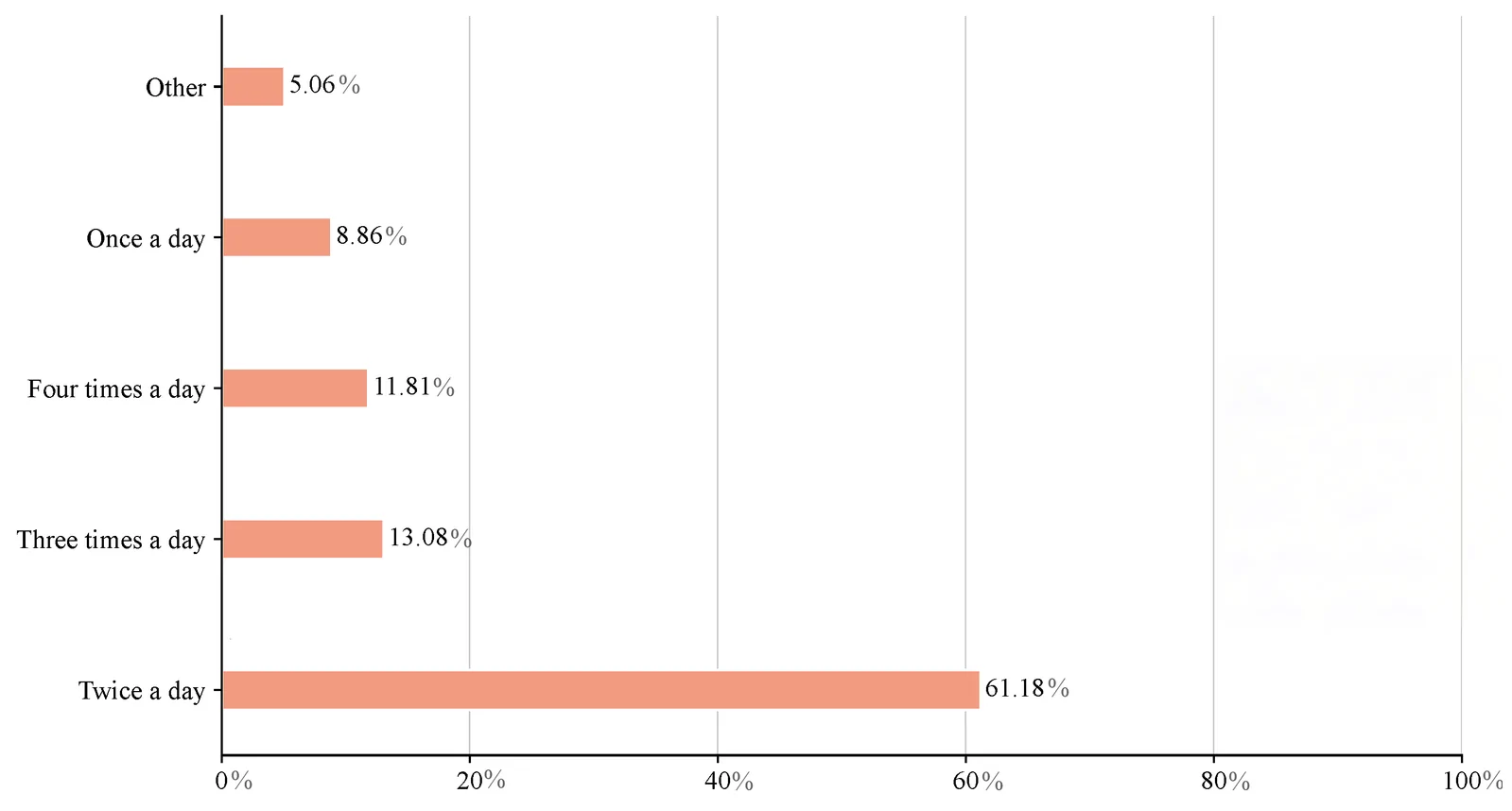

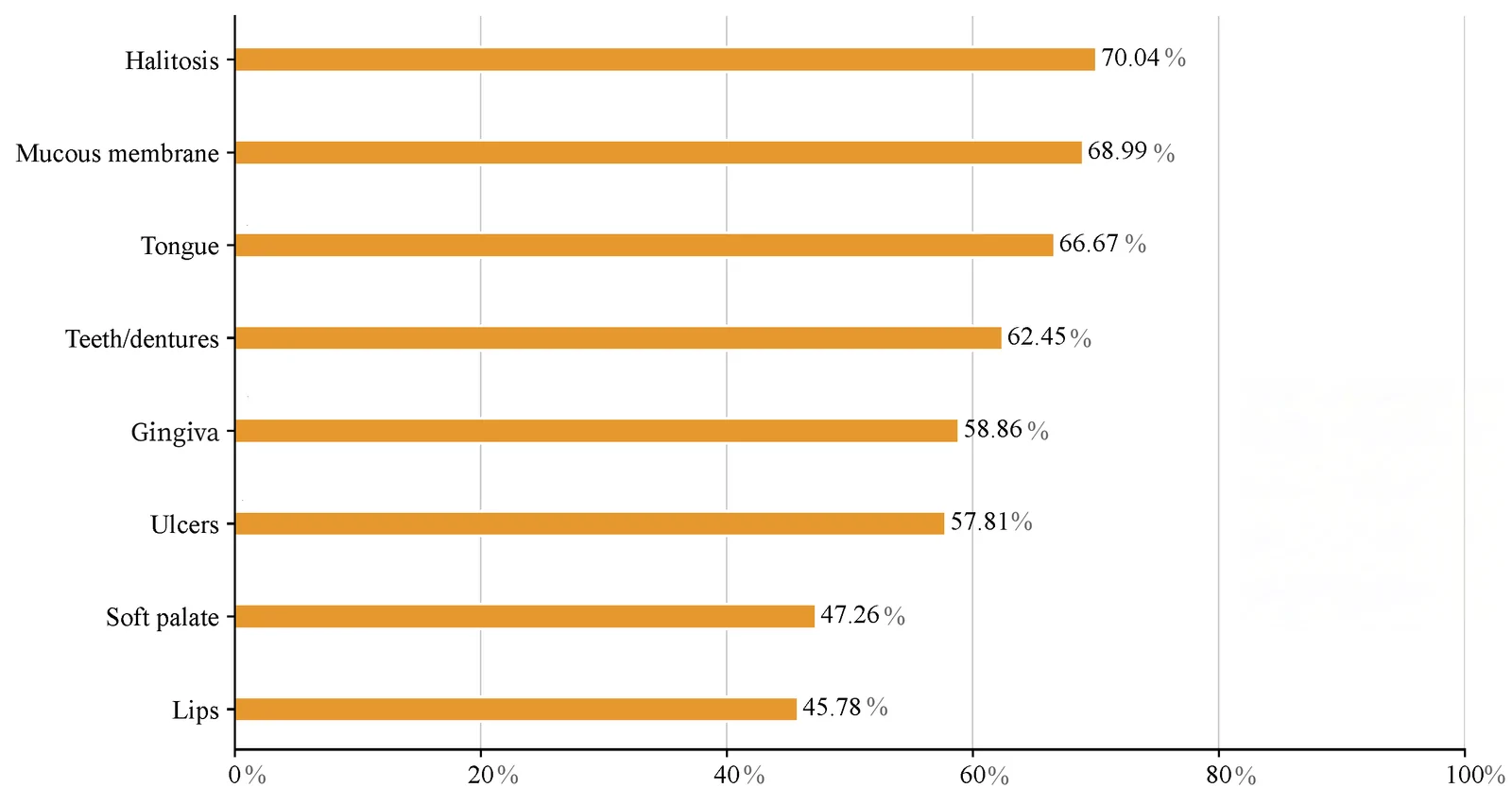

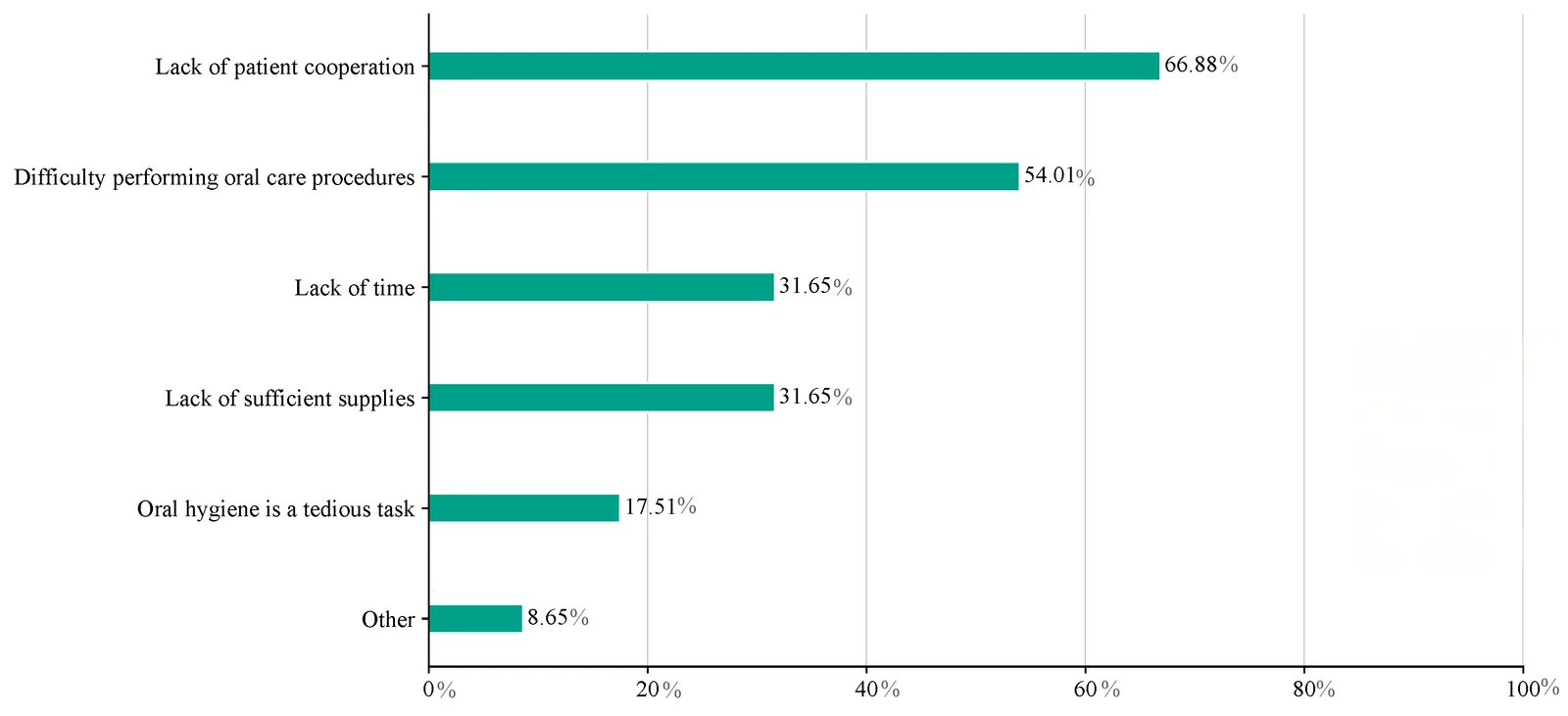

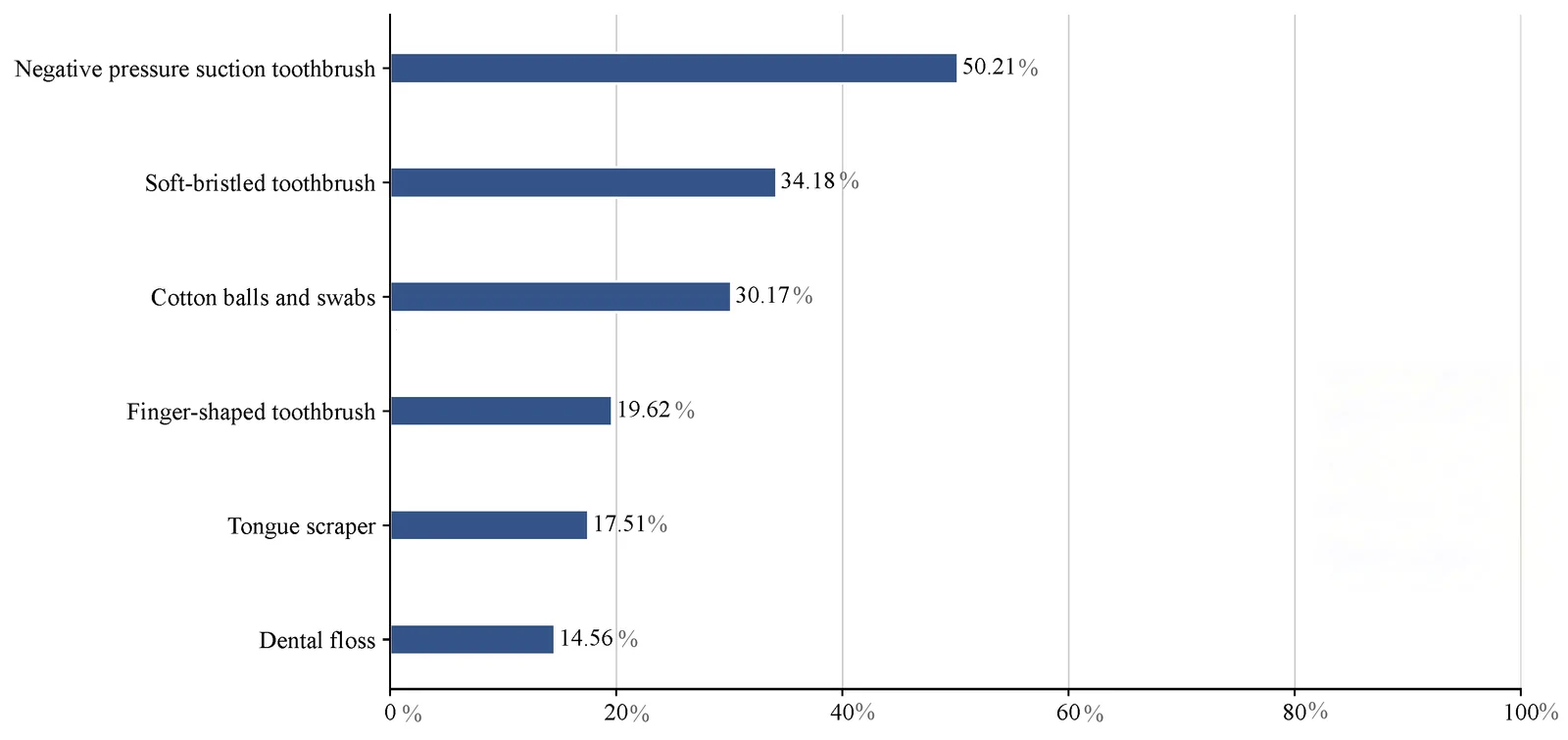

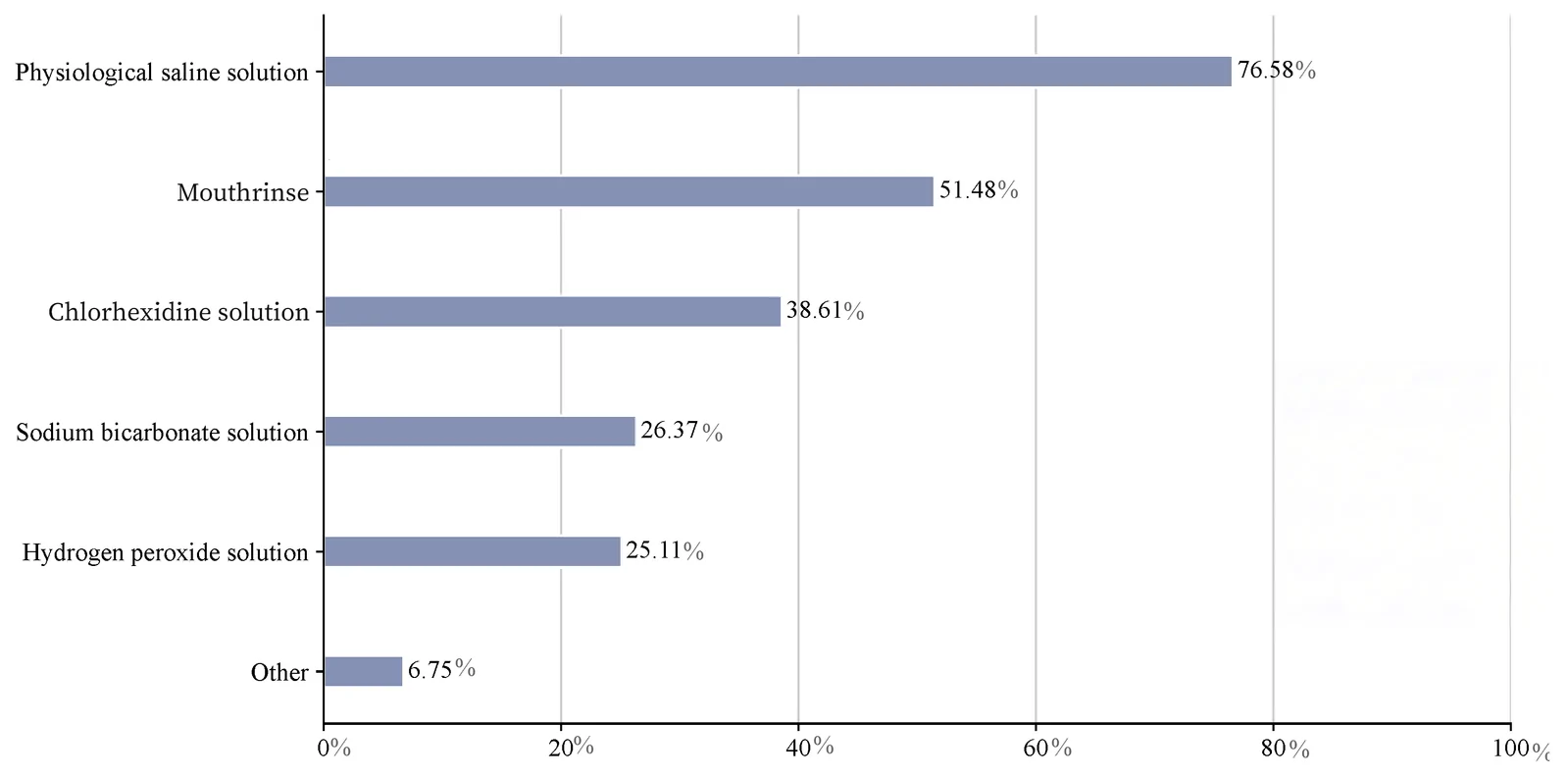
Bar charts showing neurological nurses’ responses regarding their habits and barriers to performing oral care for PSD patients

#### Univariate analysis of KAP of neurological nurses regarding oral care for PSD patients

Univariate correlation analysis revealed that hospital level, educational attainment, length of neurological ward experience, and prior specialised training had statistically significant effects on the overall KAP scores of neurological nurses providing oral care for patients with PSD (*P* < 0.05), as presented in Table 3.

**Table 3 Table3:** Univariate correlation analysis of KAP of neurological nurses regarding oral care for PSD patients

Variable		Label	Knowledge	Attitude	Practice	KAP
Hospital level	Primary	a	12.75 ± 3.22^bc^	36.33 ± 14.71^bc^	30.17 ± 10.62^bc^	79.25 ± 24.86^bc^
	Secondary	b	22.25 ± 3.40^a^	52.54 ± 9.66^a^	43.01 ± 6.13^a^	117.80 ± 14.32^a^
	Tertiary	c	24.91 ± 6.82^a^	55.36 ± 10.38^a^	44.55 ± 6.56^a^	124.82 ± 16.02^a^
	F		26.835	20.272	27.386	49.294
	*P* value		< 0.001	< 0.001	< 0.001	< 0.001
Department	Neurological	a	23.10 ± 6.48	55.50 ± 11.02^c^	44.24 ± 6.91^c^	122.84 ± 17.66^c^
	Neurosurgical	b	24.72 ± 6.47	53.54 ± 10.47^c^	43.80 ± 6.80^c^	122.06 ± 17.24^c^
	Comprehensive	c	22.87 ± 6.38	48.61 ± 11.48^ab^	40.06 ± 8.91^ab^	111.55 ± 22.13^ab^
	F		2.749	5.444	4.556	5.277
	*P* value		0.065	0.005	0.011	0.006
Sex	Male	NA	20.18 ± 8.82	62.27 ± 8.46	46.09 ± 5.65	128.55 ± 16.40
	Female	NA	23.94 ± 6.39	53.70 ± 10.93	43.57 ± 7.17	121.21 ± 18.18
	t		−1.893	2.574	1.153	1.321
	*P* value		0.059	0.010	0.250	0.188
Age (y)	< 30	a	20.00 ± 7.56^bc^	56.67 ± 12.29	43.72 ± 7.38	120.38 ± 22.34
	30~40	b	24.20 ± 6.23^a^	52.97 ± 10.82^c^	43.44 ± 7.13	120.61 ± 17.98
	> 40	c	25.11 ± 5.91^a^	58.11 ± 9.19^b^	45.03 ± 6.92	128.24 ± 12.74
	F		8.264	5.090	0.818	3.038
	*P* value		< 0.001	0.007	0.442	0.049
Years working in neurological department	≤3	a	19.06 ± 6.97^bcd^	51.32 ± 14.73	40.98 ± 10.55^bd^	111.36 ± 28.01^bcd^
	4~6	b	23.89 ± 6.60^a^	56.00 ± 10.38	44.20 ± 5.51^a^	124.08 ± 16.01^a^
	7~9	c	24.95 ± 5.03^a^	52.72 ± 11.20	42.11 ± 7.34	119.79 ± 17.55^a^
	≥ 10	d	24.71 ± 6.26^a^	54.44 ± 9.69	44.76 ± 6.11^a^	123.91 ± 14.34^a^
	F		10.943	1.999	4.772	6.803
	*P* value		< 0.001	0.114	0.003	< 0.001
Educational level	Associate degree and lower	NA	20.43 ± 7.35	50.51 ± 14.21	40.61 ± 10.59	111.55 ± 27.44
	Bachelor’s degree and above	NA	24.42 ± 6.16	54.59 ± 10.18	44.20 ± 6.20	123.21 ± 15.35
	t		−4.132	−1.964	−2.349	−2.953
	*P* value		< 0.001	0.054	0.022	0.005
Position	Nurse	NA	23.60 ± 6.58	53.60 ± 11.02	43.55 ± 7.07	120.75 ± 18.24
	Head nurse	NA	25.64 ± 5.57	57.08 ± 10.08	44.50 ± 7.71	127.22 ± 16.51
	t		−1.786	−1.806	−0.753	−2.030
	*P* value		0.075	0.072	0.452	0.043
Title	Nurse	a	20.63 ± 7.14^bc^	53.43 ± 13.31	42.20 ± 8.08	116.26 ± 23.49^c^
	Nurse practitioner	b	24.07 ± 6.44^a^	52.36 ± 11.16	43.42 ± 6.55	119.84 ± 17.67
	Head nurse and above	c	24.70 ± 6.02^a^	55.39 ± 9.76	44.32 ± 7.19	124.40 ± 15.91^a^
	F		8.407	2.752	1.887	4.921
	*P* value		< 0.001	0.065	0.153	0.008
Monthly income (Yuan)	< 6,000	a	22.33 ± 6.18^bc^	53.51 ± 11.45	43.17 ± 7.28	119.01 ± 19.01^c^
	6,000~10,000	b	25.17 ± 6.61^a^	54.03 ± 11.03	44.09 ± 7.06	123.28 ± 17.47
	> 10,000	c	26.57 ± 5.97^a^	55.83 ± 8.30	44.57 ± 6.67	126.98 ± 14.32^a^
	F		11.689	0.764	0.981	4.249
	*P* value		< 0.001	0.467	0.376	0.015
Training	Yes	NA	23.21 ± 6.28	57.92 ± 9.91	45.47 ± 6.46	126.60 ± 17.19
	No	NA	24.06 ± 6.58	52.36 ± 10.98	42.91 ± 7.28	119.33 ± 18.14
	t		−1.089	4.339	3.029	3.392
	*P* value		0.277	< 0.001	0.003	0.001
Pairwise comparisons were performed using the SNK-q test. ^a^ following a mean indicates a significant difference (*P* < 0.05) compared to level a of that variable; ^b^ indicates a significant difference (*P* < 0.05) compared to level b; ^c^ indicates a significant difference (*P* < 0.05) compared to level c. NA, not applicable.						

#### Multiple linear regression analysis of KAP of neurological nurses regarding oral care for PSD patients 

Stepwise multiple linear regression was performed, with the total KAP score of neurological nurses regarding oral care for PSD patients designated as the dependent variable, and all variables showing statistical significance in univariate analyses entered as independent variables. Variable coding was defined as follows: Hospital level: Primary hospital = 1, Secondary hospital = 2, Tertiary hospital = 3; Education level: Technical secondary school diploma = 1, Junior college = 2, Bachelor’s degree = 3, Master’s degree or above = 4; Length of neurological nursing service: < 1 year = 1, 1 to 3 years = 2, 4 to 6 years = 3, 7 to 9 years = 4, ≥ 10 years = 5; Received training: Yes = 1, No = 2. Regression results indicated that hospital level, educational background, length of neurological nursing service, and previous oral care training were significant predictors of nurses’ overall KAP scores for oral care for PSD patients (Table 4).

**Table 4 Table4:** Multiple linear regression analysis of KAP of neurological nurses regarding oral care for PSD patients

Dependent variable	Independent variables		β (95%CI)	SE	SC	t	*P* value	VIF
Knowledge^a^	Intercept		34.002 (26.774, 41.229)	3.674	NA	9.255	< 0.001	0.000
	Hospital level (ref = primary)	Secondary	18.526 (12.302, 24.750)	3.164	0.732	5.856	< 0.001	6.651
		Tertiary	20.293 (14.328, 26.257)	3.032	0.837	6.693	< 0.001	6.649
	Department (ref = comprehensive)	Neurological	3.585 (−0.280, 7.451)	1.965	0.163	1.825	0.069	3.399
		Neurosurgical	2.164 (−1.725, 6.054)	1.977	0.098	1.095	0.274	3.440
	Sex (male vs female)		8.241 (2.205, 14.278)	3.068	0.134	2.686	0.008	1.056
	Age (ref = > 40 years)	< 30 years	0.546 (−4.024, 5.117)	2.323	0.016	0.235	0.814	1.961
		30~40 years	−4.668 (−8.009, −1.326)	1.699	−0.179	−2.748	0.006	1.806
	Training (yes vs no)		5.170 (2.858, 7.483)	1.175	0.215	4.399	< 0.001	1.012
Attitude^b^	Intercept		26.766 (21.724, 31.808)	2.563	NA	10.443	< 0.001	0.000
	Hospital level (ref = primary)	Secondary	12.927 (8.369, 17.485)	2.317	0.785	5.579	< 0.001	8.129
		Tertiary	13.946 (9.524, 18.368)	2.248	0.883	6.204	< 0.001	8.330
	Department (ref = comprehensive)	Neurological	2.918 (0.361, 5.474)	1.300	0.204	2.245	0.025	3.388
		Neurosurgical	2.757 (0.178, 5.336)	1.311	0.193	2.103	0.036	3.448
	Years working in neurological department (ref = ≥ 10)	≤ 3	0.604 (−2.193, 3.401)	1.422	0.029	0.425	0.671	1.963
		4~6	−0.840 (−2.796, 1.116)	0.994	−0.045	−0.845	0.399	1.186
		7~9	−3.143 (−5.062, −1.225)	0.975	−0.170	−3.223	0.001	1.141
	Educational level (bachelor’s degree and above vs associate degree and lower)		1.121 (−1.471, 3.712)	1.317	0.056	0.851	0.395	1.803
	Training (yes vs no)		2.753 (1.202, 4.303)	0.788	0.175	3.493	0.001	1.037
Practice^c^	Intercept		15.722 (11.978, 19.466)	1.903	NA	8.260	< 0.001	0.000
	Hospital level (ref = primary)	Secondary	6.968 (3.884, 10.052)	1.568	0.464	4.444	< 0.001	8.085
		Tertiary	8.875 (5.722, 12.027)	1.602	0.617	5.538	< 0.001	8.268
	Years working in neurological department (ref = ≥ 10)	≤ 3	−3.265 (−6.413, −0.116)	1.601	−0.174	−2.040	0.042	2.009
		4~6	−0.582 (−2.51, 1.347)	0.980	−0.035	−0.593	0.553	1.198
		7~9	0.664 (−0.92, 2.249)	0.806	0.039	0.825	0.410	1.144
	Educational level (bachelor’s degree and above vs associate degree and lower)		−0.549 (−3.304, 2.206)	1.401	− 0.030	− 0.392	0.695	1.796
	Monthly income (ref = < 6,000 Yuan)	6,000~10,000	1.756 (0.131, 3.382)	0.826	0.128	2.125	0.034	1.237
		> 10,000	2.590 (0.304, 4.875)	1.162	0.132	2.229	0.027	1.259
KAP^d^	Intercept		72.145 (62.174, 82.117)	5.068	NA	14.234	< 0.001	0.000
	Hospital level (ref = primary)	Secondary	36.932 (25.896, 47.968)	5.610	0.882	6.584	< 0.001	8.186
		Tertiary	43.331 (32.599, 54.062)	5.455	1.079	7.943	< 0.001	8.426
	Department (ref = comprehensive)	Neurological	6.920 (0.738, 13.101)	3.142	0.190	2.202	0.028	3.402
		Neurosurgical	6.358 (0.130, 12.586)	3.166	0.175	2.009	0.045	3.453
	Years working in neurological department (ref = ≥ 10)	≤ 3	1.256 (−5.633, 8.146)	3.502	0.024	0.359	0.720	2.046
		4~6	0.625 (−4.253, 5.504)	2.480	0.013	0.252	0.801	1.267
		7~9	−4.150 (−8.890, 0.591)	2.410	−0.088	−1.722	0.086	1.197
	Educational level (bachelor’s degree and above vs associate degree and lower)		2.449 (−3.807, 8.704)	3.180	0.048	0.770	0.442	1.805
	Position (head nurse vs nurse)		6.616 (0.709, 12.522)	3.002	0.113	2.204	0.028	1.196
	Monthly income (ref = < 6,000 yuan)	6,000~10,000 yuan	−0.279 (−4.300, 3.742)	2.044	−0.007	−0.136	0.892	1.300
		> 10,000 yuan	1.127 (−4.658, 6.912)	2.941	0.021	0.383	0.702	1.311
	Training (yes vs no)		6.763 (3.001, 10.525)	1.912	0.169	3.536	< 0.001	1.048
^a^Model 1: F = 12.156, *P* < 0.001, R^2^ = 0.229, adjusted R^2^ = 0.210; the residual autocorrelation coefficient is 0.037 and the Durbin-Watson test yields *P* = 0.239, indicating no autocorrelation in residuals; the White test chi-square value is 36.700, *P* = 0.186, suggesting homoscedasticity of residuals. ^b^Model 2: F = 9.306, *P* < 0.001, R^2^ = 0.203, adjusted R^2^ = 0.182; the residual autocorrelation coefficient is 0.016 and the Durbin-Watson test yields *P* = 0.372, indicating no autocorrelation in residuals; the White test chi-square value is 48.311, *P* = 0.122, suggesting homoscedasticity of residuals. ^c^Model 3: F = 9.529, *P* < 0.001; R^2^ = 0.189, adjusted R^2^ = 0.169; the residual autocorrelation coefficient is −0.032 and the Durbin-Watson test yields *P* = 0.29, indicating no autocorrelation in residuals; the White test chi-square value is 62.408, *P* < 0.001, suggesting heteroscedasticity of residuals, so the heteroscedasticity-consistent covariance (HCC) estimator was applied to obtain valid t-values, *P* values, and 95% confidence intervals for the coefficient estimates. ^d^Model 4: F = 11.044, *P* < 0.001, R^2^ = 0.290, adjusted R^2^ = 0.264; the residual autocorrelation coefficient is 0.045 and the Durbin-Watson test yields *P* = 0.196, indicating no autocorrelation in residuals; the White test chi-square value is 65.735, *P *= 0.589, suggesting homoscedasticity of residuals. NA, not applicable; VIF, variance inflation factor; all VIF values were < 10, indicating no multicollinearity among the independent variables.								

## Discussion

### The Knowledge Dimension

Neurological nurses exhibited deficiencies in professional knowledge related to oral care for patients with PSD. The knowledge dimension score in this study was slightly lower than that reported by Xu et al.^29^ This discrepancy may stem from the difference in sampling: the present study recruited nurses from primary, secondary, and tertiary hospitals, whereas Xu et al^29^ only surveyed staff working in tertiary hospitals.The three lowest-scoring items were ‘concentration of chlorhexidine’, ‘Bass brushing technique’, and ‘medications causing dry mouth’. First, most nurses mainly rely on normal saline as an oral cleaning solution, which can be attributed to their insufficient knowledge of the physicochemical properties, applicable concentrations and adverse effects of various oral cleaning solutions. In the present sample, scores for knowledge about the chlorhexidine solution were only 3.623 ± 1.112, which is consistent with the studies of Asadi and Jahanimoghadam.^2^ Secondly, standardised brushing techniques and knowledge of xerostomia-inducing medications are frequently overlooked, largely owing to insufficient cross-disciplinary training programmes for neurological nurses. In this study, only 29.08% of participants had received training on oral care. This aligns with a study by Bossouf et al,^6^ who reported that only 35.8% of nurses in France had received relevant training. This lack of the knowledge dimension indicates a need for greater emphasis on professional development, especially with therapists, dietitians, and dental professionals. Ozaki et al^21^ demonstrated that the suppression of SAP is effectively achieved through early intervention and education of nurses by dental professionals.^18,23
^ However, multidisciplinary teams for dysphagia emphasise collaboration with therapists and dietitians, with limited involvement from dental professionals currently. Hospital administrators should therefore prioritise interdisciplinary collaboration with dental professionals and adopt cross-disciplinary training programmes to improve nurses’ oral care knowledge base.

### The Attitudes Dimension

This study revealed that nurses’ attitudes towards PSD oral care also had prominent deficiencies, despite them displaying basic recognition of its importance. Items concerning identification of oral infectious signs and formulation of ward-specific oral care procedures obtained low scores, reflecting two core attitudinal defects. Items such as ‘Do you think you know how to identify and prevent oral infections?’ and ‘Do you think the department should formulate and update oral care plans?’ scored relatively low. The relatively low scores in identifying and preventing oral infections suggest a lack of related professional knowledge among nurses. Similarly, the low approval rate for developing and updating oral care plans reflects deficiencies in the institutionalisation and standardisation of management within the department. The lack of standardised guidelines hinders departments’ attention to formulating and updating oral care programmes for PSD patients. Although the Registered Nurses’ Association of Ontario^22^ has released oral care best practices suggestions for specific populations such as PSD patients, many countries and regions, including China, lack similar localised guidelines and expert consensus. The absence of standardised oral care procedures leads to oral care being neglected for PSD patients. In this study, the item ‘Oral care is relatively easy to neglect’ scored 4.08 ± 0.807, consistent with the results reported by Asadi et al.^2^. This finding demonstrates that oral care often is frequently deprioritised relative to other clinical responsibilities. To address this gap, it is recommended to increase original research focused on oral care for patients with PSD and to promote the development of relevant guidelines and expert consensus. Such interventions can strengthen nurses’ awareness, knowledge, and clinical practices, thereby improving oral hygiene status and overall rehabilitation outcomes among PSD patients.

### The Practices Dimension

The practices dimension showed suboptimal performance, demonstrating that participants failed to translate limited theoretical knowledge and inadequate positive attitudes into standardised clinical oral care practices. The lowest-scoring items revealed that neurological nurses were rarely engaged in routine oral assessments and seldom guided patients to receive oral sensory and motor swallowing rehabilitation training during oral care. The results for the item ‘Daily assessment of patients’ oral conditions’ were consistent with the findings reported by Xu et al,^29^ who stressed that oral assessments should be performed for all hospitalised stroke patients. Among the multiple-choice items in the practices dimension, patient non-cooperation was identified as the leading barrier to delivering oral care, which differs from Konadu et al,^16^ who cited inadequate equipment and supplies as the top challenge. The second major obstacle to nurses providing effective oral care was the complexity of oral care procedures, consistent with the results reported by Odgaard et al.^20^ These barriers arise from two key clinical factors: first, aphasia and cognitive impairment commonly seen in patients with PSD, often result in poor patient cooperation, and second, oral dysfunction increases the complexity of oral care operations. For oral cleaning tools, suction toothbrushing was the preferred option among participants, as this guideline-endorsed approach produces better clinical results. However, nearly 30% of participants still resorted to conventional oral care approaches, a phenomenon potentially attributable to insufficient professional knowledge or patients’ financial limitations. Clinical managers should attach greater importance to standardised oral assessment, scale up oral health training and publicity, and accelerate the formulation of specialised oral care procedures and innovative techniques tailored to patients with PSD to tackle their complicated oral health issues. In parallel, relevant policymakers are advised to roll out financial support policies to guarantee adequate supply of oral cleaning tools and supplies.

### Influencing Factors of KAP Scores 

Hospital level, education level , years of clinical practice in neurology, and prior training exerted significant effects on nurses’ KAP scores related to oral care for PSD patients in the present investigation.

Neurological nurses working in tertiary hospitals achieved higher overall KAP scores. This disparity can be attributed to more standardised and systematic professional training available at tertiary institutions, covering oral care theories, practical skills, and specialised knowledge concerning dysphagia management. This study confirmed that such training equips nurses with a stronger foundation in KAP. Furthermore, tertiary hospitals are equipped with more abundant hardware and software clinical resources. As reported previously,^28^ clear role delineation within tertiary ward teams allows nurses sufficient time to deliver high-quality oral care and patient health education. In addition, nurses employed at tertiary institutions generally possess higher academic credentials and professional titles, which further improves their capacity for evidence-based clinical judgment.

Education level was another significant predictor of nurses’ KAP scores. Nurses holding a master’s degree or above receive more rigorous theoretical and hands-on training, which cultivates superior professional competency. Consistent with Asadi and Jahanimoghadam,^2^ nurses’ mastery of oral care knowledge is strongly correlated with their education level. Similarly, the present study observed that neurological nurses with higher academic credentials attained elevated KAP scores. This gap may stem from their stronger capacity to observe, reflect on, and summarise clinical problems during routine work, ultimately resulting in better KAP performance. In addition, nurses with advanced academic degrees generally have solid research capabilities, which exert a favourable effect on their clinical attitudes and practice compliance. Parallel findings were documented by Wang et al,^25^ who similarly noted higher KAP levels among nurses with master’s degrees or higher.

Years of clinical practice in neurology also positively influenced KAP scores. Participants with more than 10 years of work experience presented superior knowledge and clinical practices regarding oral care for patients with PSD, which aligns with the results reported by Chen et al^8^ and Wang et al.^25^ Oral care management for PSD patients involves complicated nursing procedures, while relevant oral care content is rarely covered in fundamental nursing education. This highlights that clinical experience is indispensable for cultivating specialised competency in this field. As nurses accumulate more clinical experience, they build in-depth insights into swallowing dysfunction, enabling them to deliver standardised and effective oral care. Furthermore, senior nurses tend to possess more proficient communication and assessment skills, allowing smoother interdisciplinary collaboration with neurologists and dental practitioners.

This study revealed that neurological nurses who received specialised training in oral care for patients with PSD attained significantly higher total KAP scores. This finding differs from the results reported by Bossouf et al,^6^ which revealed no statistically significant intergroup differences in oral care knowledge between trained and untrained nurses. Such inconsistency across studies can be explained by disparities in national nursing training systems offered in different countries. In China, nursing education institutions and pre-employment training programmes offer limited instruction in oral care, focusing primarily on basic procedures. Nevertheless, PSD patients suffer from complex oral complications that demand nurses to continuously refresh their professional knowledge and upgrade relevant practical skills. Targeted professional training deepens nurses’ recognition of the vital role played by oral care in patient rehabilitation, fosters favourable shifts in their attitudes and clinical practices, and equips them with specialised capabilities to meet the unique oral care demands of this patient group.^10^ Campbell et al^7^ demonstrated that a 1-hour training session conducted by well-trained oral health professionals can significantly improve nurses’ knowledge and attitudes towards oral care. To address these needs, nursing managers should develop comprehensive oral care management plans and implement dental professional-led training programmes specifically designed for neurological nurses responsible for PSD patient oral care.

## Conclusions

This cross-sectional survey recruited 337 neurological nurses across Southwest China via convenience sampling to explore their KAP for oral care for patients with PSD. Overall, nurses exhibited obvious deficiencies in all three KAP dimensions related to PSD oral care. In clinical practice, nurses primarily relied on saline as an oral cleaning solution and favoured negative-pressure flushing tools for oral care; patient uncooperativeness and the complexity of oral nursing procedures were identified as the top two obstacles to standard oral care delivery. Hospital level, educational level, years of clinical practice in neurology, and prior specialised oral training were confirmed as key predictors of nurses’ overall KAP levels. To narrow the gaps in oral care competency, primary and secondary medical institutions should carry out regular academic exchanges and skill-sharing with tertiary hospitals. Nursing administrators ought to build stable multidisciplinary collaboration mechanisms with dental departments. Targeted oral care training programmes and standardised operating protocols should also be formulated in accordance with nurses’ educational backgrounds and working seniority to comprehensively improve their oral care knowledge, awareness, and clinical practice capacity for PSD patients.

### Limitations of this Study

This study recruited participants via convenience sampling from Southwest Chinese public hospitals, with several inherent limitations. First, the regression model for total KAP scores had a low coefficient of determination (R^2^ = 0.223), showing limited explanatory power. Nurses’ oral care performance is affected by unmeasured latent variables including ward organisational culture, clinical workload, and self-efficacy. Future studies should incorporate more dimensional predictors or adopt multilevel linear modelling to improve model explanatory capacity. Second, the sample distribution across hospital tiers was highly unbalanced (72.11% tertiary vs only 2.37% primary), resulting in selection bias. Tertiary hospital nurses have richer clinical resources and standardised training, so the results cannot fully represent grassroots medical staff; community hospitals, rehabilitation centres, and nursing homes were excluded from sampling. Third, only neurological nurses were surveyed, limiting the generalisability to staff managing intensive care unit–acquired or age-related dysphagia. Self-reported questionnaires may also introduce subjective response bias.

For future research, stratified random sampling is recommended to expand regional and institutional coverage and increase the proportion of primary hospital participants. Combined objective clinical assessment tools and questionnaires can more accurately clarify variable correlations.

This work was funded by Chongqing Municipal Sports Bureau Projects, B2024101.

## References

## References

[ref1] Adams R (1996). Qualified nurses lack adequate knowledge related to oral health, resulting in inadequate oral care of patients on medical wards. J Adv Nurs.

[ref2] Asadi N, Jahanimoghadam F (2024). Oral care of intubated patients, challenging task of ICU nurses: A survey of knowledge, attitudes and practices. BMC Oral Health.

[ref3] BaHammam F, Durham J, Abdulmohsen B, Wassall R, McCracken GI (2023). Oral health decline in patients after stroke: A qualitative study. Br Dent J.

[ref4] Bangee M, Martinez-Garduno CM, Brady MC (2021). Oral care practices in stroke: Findings from the UK and Australia. BMC Nurs.

[ref5] Binkley C, Furr LA, Carrico R, McCurren C (2004). Survey of oral care practices in US intensive care units. Am J Infect Control.

[ref6] Bossouf A, Sabourin C, Pop C, Giraudeau N, Inquimbert C (2023). Interprofessional survey on knowledge and attitudes on oral health among nurses in France. Oral Health Prev Dent.

[ref8] Chen J, Ye W, Zheng X, Wu W, Chen Y, Chen Y (2024). Predictors of medical staff’s knowledge, attitudes and behavior of dysphagia assessment: A cross-sectional study. PLoS One.

[ref9] Chinese Association of Rehabilitation Medicine and Dysphagia Rehabilitation Professional Committee (2023). Chinese Dysphagia Rehabilitation Management Guidelines (2023 Edition). Chinese Journal of Physical Medicine and Rehabilitation.

[ref10] Curtin C, Barrett A, Burke FM, McKenna G, Healy L, Hayes M (2024). Exploring facilitators and barriers associated with oral care for inpatients with dysphagia post-stroke. Gerodontology.

[ref11] Dibello V, Lobbezoo F, Lozupone M (2023). Oral frailty indicators to target major adverse health-related outcomes in older age: A systematic review. Geroscience.

[ref14] Furuya J, Suzuki H, Tamada Y (2020). Food intake and oral health status of inpatients with dysphagia in acute care settings. J Oral Rehabil.

[ref15] Huang YT, Tang CC, Chung CC, Chung CH (2025). Effects of oral care combined with neuromuscular electrical stimulation on clinical outcomes in the acute phase of acute ischemic stroke: A pilot randomized controlled trial. J Neuroeng Rehabil.

[ref16] Konadu AB, Iwuozo EU, Sunkwa-Mills G (2024). Healthcare professionals’ perspectives on oral health care in acute stroke patients: A qualitative study. BDJ Open.

[ref18] Labeit B, Michou E, Hamdy S (2023). The assessment of dysphagia after stroke: State of the art and future directions. Lancet Neurol.

[ref19] Lin YS, Chang JC, Chang TH, Lou MF (2011). Critical care nurses’ knowledge, attitudes and practices of oral care for patients with oral endotracheal intubation: A questionnaire survey. J Clin Nurs.

[ref20] Odgaard L, Kothari M (2019). Survey of oral nursing care attitudes, knowledge and practices in a neurorehabilitation setting. J Oral Rehabil.

[ref21] Ozaki K, Tohara H, Baba M, Teranaka S, Kawai Y, Komatsumoto S (2023). A dentist-led oral care system can prevent stroke-associated pneumonia: The effects of early intervention by dental team. J Multidiscip Healthc.

[ref23] Shimizu A, Ohno T, Fujishima I (2023). Impact of poor oral health status on swallowing function improvement in older dysphagic patients. Cureus.

[ref24] Tian F, Li J, Wu B (2023). Differences in the oral health status in hospitalised stroke patients according to swallowing function: A cross-sectional study. J Clin Nurs.

[ref25] Wang R, Song Y, He Y, Long S, Feng L (2023). Status of knowledge, attitude and practice of poststroke dysphagia in neurological nurses in China: A cross-sectional study. PLoS One.

[ref27] Wu C, Huang H, Xu W, Li J, Chen M, Zhao Q (2023). Influencing factors associated with oral health among older hospitalized patients with ischemic stroke: A cross-sectional survey. Int J Nurs Sci.

[ref28] Xiang Y, Liu J, Liu X, Wang Y, You L (2021). Analysis of nurses work status and differences between secondary and tertiary hospitals in Guangdong Province. Chin J Nurs.

[ref29] Xu Y, Zhou X, Wei J (2024). Analysis of clinical nurses’ knowledge, attitude and practice and the influencing factors of hospitalized stroke patient’s oral nursing. Nurs Pract Res.

[ref30] Yang S, Park JW, Min K (2023). Clinical practice guidelines for oropharyngeal dysphagia. Ann Rehabil Med.

